# Supplementary material for: Information Technology Ambidexterity, Digital Dynamic Capability, and Knowledge Processes as Enablers of Patient Agility: Empirical Study
Source: JMIRx Med. 2021 Dec 6;2(4):e32336. doi: 10.2196/32336 (PMC10414313; doi:10.2196/32336)
Supplement: Multimedia Appendix 1 [file xmed_v2i4e32336_app1.docx]

## Survey constructs and items and descriptive statistics

| **Construct** | | **Measurement item** | ***λ*** | ***μ*** | **Std.** |
| --- | --- | --- | --- | --- | --- |
| IT ambidexterity | *Please indicate the ability of your department to: (1. Strongly disagree–7. Strongly agree)* | | | | |
|  | *IT exploration capability* | |  |  |  |
|  | EXPLR1 | Acquire new IT resources (e.g., potential IT applications, critical IT skills) | 0.860 | 4.01 | 1.67 |
|  | EXPLR2 | Experiment with new IT resources | 0.915 | 3.81 | 1.62 |
|  | EXPLR3 | Experiment with new IT management practices | 0.889 | 3.43 | 1.62 |
|  | *IT exploitation capability* | |  |  |  |
|  | EXPLO1 | Reuse existing IT components, such as hardware and network resources | 0.921 | 5.29 | 1.28 |
|  | EXPLO2 | Reuse existing IT applications and services | 0.944 | 5.18 | 1.32 |
|  | EXPLO3 | Reuse existing IT skills | 0.944 | 5.13 | 1.25 |
| *Dig. dynamic capability* | *Please indicate the level of your department’s capabilities in following areas (1. Strongly disagree–7. Strongly agree).* | | | | |
|  | DDC1 | Responding to digital transformation | 0.886 | 4.33 | 1.56 |
|  | DDC2 | Mastering the state-of-the-art digital technologies | 0.895 | 3.69 | 1.48 |
|  | DDC3 | Developing innovative patient services using digital technology | 0.856 | 4.74 | 1.63 |
| *Patient agility* | *Indicate the degree to which you agree or disagree with the following statements about whether the department can (1 – strongly disagree 7 – strongly agree)* | | | | |
|  | *Patient sensing capability* | |  |  |  |
|  | SENSE1 | We continuously discover additional needs of our patients of which they are unaware | 0.884 | 4.09 | 1.66 |
|  | SENSE2 | We extrapolate key trends for insights on what patients will need in the future | 0.760 | 4.43 | 1.63 |
|  | SENSE3 | We continuously anticipate our patients’ needs even before they are aware of them | 0.893 | 4.03 | 1.68 |
|  | SENSE4 | We attempt to develop new ways of looking at patients and their needs | 0.791 | 4.72 | 1.52 |
|  | SENSE5 | We sense our patient’s needs even before they are aware of them | 0.868 | 3.94 | 1.66 |
|  | *Patient responding capability* | |  |  |  |
|  | RESPOND1 | We respond rapidly if something important happens with regard to our patients | 0.935 | 4.76 | 1.71 |
|  | RESPOND2 | We quickly implement our planned activities with regard to patients | 0.918 | 4.11 | 1.62 |
|  | RESPOND3 | We quickly react to fundamental changes with regard to our patients | 0.917 | 4.54 | 1.53 |
|  | RESPOND4 | When we identify a new patient need, we are quick to respond to it | 0.868 | 4.52 | 1.42 |
|  | RESPOND5 | We are fast to respond to changes in our patient’s health service needs | 0.865 | 4.52 | 1.50 |
| *Knowledge processes* | *Indicate the degree to which you agree or disagree with the following statements about whether the department can (1 – strongly disagree 7 – strongly agree)* | | | | |
|  | KP1 | We regularly meet patients to learn about their current and potential needs for new health services | 0.676 | 3.99 | 1.64 |
|  | KP2 | Our knowledge of patients’ needs is thorough | 0.810 | 4.10 | 1.69 |
|  | KP3 | We systematically process and analyze patient data and information | 0.715 | 4.59 | 1.67 |
|  | KP4 | We regularly study our patient’s needs for new health service development | 0.790 | 4.02 | 1.53 |
|  | KP5 | We have interdepartmental meetings regularly to discuss patient’s needs | 0.857 | 4.04 | 1.71 |
|  | KP6 | Our department spend time discussing patient’s future needs with other (clinical) departments | 0.838 | 3.97 | 1.65 |
