# Supplementary material for: Information Technology Ambidexterity, Digital Dynamic Capability, and Knowledge Processes as Enablers of Patient Agility: Empirical Study
Source: JMIRx Med. 2021 Dec 6;2(4):e32336. doi: 10.2196/32336 (PMC10414313; doi:10.2196/32336)
Supplement: Multimedia Appendix 2 [file xmed_v2i4e32336_app2.docx]

## Survey response per medical department

| **Department** | **# responses** | **% of total** |
| --- | --- | --- |
| General Internal Medicine | 3 | 3% |
| Anesthesiology | 4 | 4% |
| Pharmacy | 2 | 2% |
| Cardiology | 7 | 7% |
| Cardiothoracic surgery | 2 | 2% |
| Surgery | 8 | 7% |
| Dermatology | 3 | 3% |
| Endocrinology | 1 | 1% |
| Geriatrics | 1 | 1% |
| Infectious diseases | 1 | 1% |
| Intensive Care Adults | 5 | 5% |
| Pediatrics | 8 | 7% |
| Neonatology | 2 | 2% |
| Clinical immunology & Rheumatology | 2 | 2% |
| Clinical Oncology | 2 | 2% |
| Lung diseases | 2 | 2% |
| Gastrointestinal and liver diseases | 4 | 4% |
| Neurosurgery | 2 | 2% |
| Neurology | 4 | 4% |
| Kidney diseases | 3 | 3% |
| Ophthalmology | 2 | 2% |
| Orthopedics | 5 | 5% |
| Psychiatry | 2 | 2% |
| Revalidation | 1 | 1% |
| First aid | 6 | 6% |
| Urology | 3 | 3% |
| Vascular medicine | 2 | 2% |
| Obstetrics / Gynecology | 9 | 8% |
| Medical imaging | 6 | 6% |
| Day treatment | 3 | 3% |
| Radiotherapy | 1 | 1% |
| Paramedic care | 1 | 1% |
| **Total** | **107** | **100%** |
