# Supplementary material for: Information Technology Ambidexterity, Digital Dynamic Capability, and Knowledge Processes as Enablers of Patient Agility: Empirical Study
Source: JMIRx Med. 2021 Dec 6;2(4):e32336. doi: 10.2196/32336 (PMC10414313; doi:10.2196/32336)
Supplement: Multimedia Appendix 3 [file xmed_v2i4e32336_app3.docx]

## Cross-loading analysis for the first-order factors

|  | EXPLR | EXPLO | DDC | PSC | PSR | KP |
| --- | --- | --- | --- | --- | --- | --- |
| Explore1 | **0.921** | 0.487 | 0.543 | 0.345 | 0.211 | 0.418 |
| Explore2 | **0.944** | 0.454 | 0.494 | 0.238 | 0.239 | 0.338 |
| Explore3 | **0.944** | 0.419 | 0.529 | 0.356 | 0.350 | 0.413 |
| Exploit1 | 0.514 | **0.860** | 0.578 | 0.403 | 0.379 | 0.442 |
| Exploit2 | 0.397 | **0.915** | 0.575 | 0.447 | 0.283 | 0.403 |
| Exploit3 | 0.374 | **0.889** | 0.513 | 0.440 | 0.214 | 0.420 |
| Dig1 | 0.441 | 0.529 | **0.886** | 0.436 | 0.422 | 0.432 |
| Dig2 | 0.547 | 0.526 | **0.895** | 0.506 | 0.411 | 0.486 |
| Dig3 | 0.480 | 0.595 | **0.856** | 0.525 | 0.441 | 0.467 |
| Sense1 | 0.381 | 0.481 | 0.507 | **0.884** | 0.473 | 0.645 |
| Sense2 | 0.484 | 0.512 | 0.502 | **0.760** | 0.346 | 0.560 |
| Sense3 | 0.261 | 0.357 | 0.463 | **0.893** | 0.552 | 0.593 |
| Sense4 | 0.113 | 0.409 | 0.455 | **0.791** | 0.372 | 0.545 |
| Sense5 | 0.191 | 0.302 | 0.432 | **0.868** | 0.452 | 0.560 |
| Respond1 | 0.216 | 0.299 | 0.405 | 0.481 | **0.935** | 0.294 |
| Respond2 | 0.239 | 0.300 | 0.442 | 0.436 | **0.918** | 0.240 |
| Respond3 | 0.310 | 0.312 | 0.412 | 0.444 | **0.917** | 0.321 |
| Respond4 | 0.292 | 0.356 | 0.503 | 0.498 | **0.868** | 0.352 |
| Respond5 | 0.227 | 0.230 | 0.416 | 0.520 | **0.865** | 0.500 |
| KP1 | 0.277 | 0.266 | 0.193 | 0.361 | 0.164 | **0.676** |
| KP2 | 0.337 | 0.372 | 0.481 | 0.596 | 0.339 | **0.810** |
| KP3 | 0.389 | 0.275 | 0.395 | 0.516 | 0.304 | **0.715** |
| KP4 | 0.314 | 0.412 | 0.412 | 0.461 | 0.099 | **0.790** |
| KP5 | 0.357 | 0.404 | 0.476 | 0.581 | 0.320 | **0.857** |

*Note: EXPLR: IT exploration EXPLO: IT exploitation; DDC: digital dynamic capability; PSC: patient sensing capability; PRC: patient responding capability; KP: knowledge processes*
